# Characterization of Food-Additive Titanium Dioxide and Dietary Exposure to Titanium Dioxide Nanoparticles among the Chinese Population

**DOI:** 10.3390/nano14171427

**Published:** 2024-08-31

**Authors:** Yue Cao, Huali Wang, Chunlai Liang, Qing Liu, Tong Ou, Ling Yong, Xiao Xiao, Haixia Sui, Dingguo Jiang, Zhaoping Liu, Sheng Wei, Yan Song

**Affiliations:** 1 Key Laboratory of Food Safety Risk Assessment, China National Center for Food Safety Risk Assessment, Guangqu Road, Beijing 100022, China; m202175501@hust.edu.cn (Y.C.); wanghuali@cfsa.net.cn (H.W.); liangchunlai@cfsa.net.cn (C.L.); liuqing@cfsa.net.cn (Q.L.); outong@cfsa.net.cn (T.O.); yongling@cfsa.net.cn (L.Y.); xiaoxiao@cfsa.net.cn (X.X.); suihaixia@cfsa.net.cn (H.S.); jiangdingguo@cfsa.net.cn (D.J.); liuzhaoping@cfsa.net.cn (Z.L.); 2 School of Public Health, Tongji Medical College, Huazhong University of Science and Technology, Hangkong Road, Wuhan 430030, China; 3 School of Public Health and Emergency Management, Southern University of Science and Technology, 1088 Academy Avenue, Nanshan District, Shenzhen 518055, China

**Keywords:** food-additive titanium dioxide, nanoparticles, characterization, dietary exposure, Chinese population

## Abstract

Titanium dioxide (TiO_2_) is a prevalent food additive, yet comprehensive data on particle size and dietary exposure are lacking in China. Transmission electron microscopy results revealed that the quantitative proportion of nanoparticles (NPs) in food-additive TiO_2_ was 37.7%, with a mass fraction of 9.89%. Laboratory test results showed that among the domestic products surveyed, candies excluding gum-based candies contained the highest content of TiO_2_. Using consumption data from the China Health and Nutrition Survey in 2018, the average dietary exposure for TiO_2_ and TiO_2_ NPs in the Chinese population were calculated at 34.84 and 3.44 μg/kg bw/day, respectively. The primary dietary sources were puffed food and powdered drinks. Exposure varied significantly across age and region, with children and Inner Mongolia residents having the highest intake. TiO_2_ NP exposure showed a negative correlation with age. Despite this, the dietary exposure risk of TiO_2_ NPs for the Chinese population remains deemed acceptable.

## 1. Introduction

Titanium dioxide (TiO_2_) is widely used in various applications, including food, cosmetics, pharmaceuticals, and paints, primarily due to its coloring, photocatalytic, and biocidal properties [1,2]. The European Union refers to food-additive TiO_2_ as E171. E171 is found in higher concentrations in foods such as chewing gum, candies, and puddings, as it is commonly used as a coating for confectionery products [3,4,5]. Nevertheless, recent years have seen a notable decline in its utilization, partly due to growing concerns regarding its safety [6].

Studies have suggested that nanoparticle (NPs) within TiO_2_ particles may pose potential risks to human health. The nature and magnitude of the damage caused by TiO_2_ NPs are influenced by their physical and chemical properties. Additionally, the reactivity and bioavailability of TiO_2_ NPs are determined by these specific characteristics [7,8]. Oral exposure to E171 can alter the gut microbiota of experimental animals, leading to changes in colon pH, significantly increasing oxidative stress and inflammatory responses [9,10]. Multiple studies have demonstrated that following oral or inhalation exposure to TiO_2_ NPs, accumulation can occur in vital organs such as the gastrointestinal system, lungs, heart, and liver [11,12]. Furthermore, TiO_2_ NPs have the potential to induce genotoxic effects including DNA damage or chromosomal instability [13,14,15], as well as cytotoxicity [16]. This is mainly due to the production of reactive oxygen species, which leads to cellular damage, inflammation, genotoxicity, and adverse immune responses [17,18,19,20]. 

Consequently, regulatory agencies and industries in various nations have actively engaged in assessing and addressing the concerns surrounding the use and safety of TiO_2_. In 2021, the European Food Safety Authority (EFSA) released a report concluding that TiO_2_ particles may induce DNA strand breaks and chromosomal damage, although they did not induce gene mutations. Consequently, the use of E171 as a food additive is no longer considered safe, and the sale of foods containing TiO_2_ is banned in Europe [21]. However, entities such as the Health Canada [22] and the UK Food Standards Agency [23] reviewed related research and found no evidence to suggest that dietary intake of food-grade TiO_2_ was harmful to human health. Existing domestic research offers limited and outdated data on the systematic assessment of dietary intake of TiO_2_. 

In this study, electron microscopy was utilized to characterize and examine food-additive TiO_2_ to obtain the percentage of NPs in both quantity and mass. The TiO_2_ content in various food categories was determined using detection methods compliant with national food safety standards. The study aims to provide the most recent dietary exposure assessment results for TiO_2_ and TiO_2_ NPs in the Chinese population with updated consumption survey data. The differences in population characteristics and primary dietary sources were further identified. These findings are crucial for improving the regulatory framework for food-additive TiO_2_ and prioritizing the protection of high-risk populations.

## 2. Materials and Methods

### 2.1. Study Design

This study chose food-additive TiO_2_ samples available in domestic markets and analyzed particle size using electron microscopy. Statistical software and calculation formulas were employed to determine the numerical percentage and mass fractions of NPs. The TiO_2_ content in food products sold in various provinces in China was detected using standard laboratory methods. In conjunction with consumption data from the Chinese Resident Food Consumption Survey in 2018, the daily exposure levels of TiO_2_ in the Chinese population were assessed. The exposure risk of TiO_2_ NPs was determined by multiplying the mass fraction of NPs. Furthermore, population characteristic differences were used to identify high-risk age groups, genders, and regions. Contribution rates of different food categories were analyzed to confirm the primary dietary sources.

### 2.2. Particle Size Distributions of Food-Additive TiO_2_ by Transmission Electron Microscopy (TEM)

TiO_2_ samples were from two leading companies in China that produce food-additive TiO_2_: Jiangsu Hushen Titanium White Technology Co., Ltd. (Jiangsu, China) and Tianjin Duofuyuan Industrial Co., Ltd. (Tianjin, China). A total of 10 samples from different production batches of each company were randomly selected. Additionally, a sample was provided by the China National Center for Food Safety Risk Assessment. The sources and batch numbers of the samples are specified in Table S1.

The TiO_2_ powder was weighed to a precise mass of 10.00 ± 0.25 mg for each sample, followed by its transformation into a TiO_2_ suspension through ultrasonic treatment. The size and morphology of the particles were characterized using TEM (model HT7700, Hitachi, Japan). TEM images of TiO_2_ were obtained at parameters of an accelerating voltage of 10.0 kV, a working distance of 500 nm, and a magnification of 20,000. The procedure for the preparation of samples and the detection of particle size for food-additive TiO_2_ is shown in Supplementary Material S1. The TEM images of maximum and minimum Feret diameters measured in the TiO_2_ samples are shown in Figure S1.

To determine the quantitative proportions of NPs, the percentage of TiO_2_ particles with a minimum Feret diameter smaller than 100 nm was calculated from the TEM image for each sample, using the ImageJ 1.53t software. Following the analysis methods for the identification and characterization of NPs in food additives developed by EFSA [24], we further determined the mass fraction of NPs. The short (a-axis) and long axes (c-axis) of the ellipse fitted to the 2D projection of each constituent particle were measured as proxies of their minimum and maximum Feret dimensions, respectively. Assuming that the particles are prolate ellipsoids (*a*-axis = *b*-axis < c-axis), the volume (*V*) of each particle was estimated as:

(1) $$V=\frac{4}{3}\pi a^{2}c$$

The mass (*M*) of each particle was calculated as:

(2) M=V×ρ

*ρ* = the calculated bulk density of anatase TiO_2_ = 3.89 g cm^−3^ (the crystal type of the TiO_2_ samples supplied was anatase).

The mass fraction (*M%*) was given by the following formula:

(3) $$M\%=M_{NPs}÷M_{TiO2}$$

*M_NPs_* is the mass of NPs and *M_TiO2_* is the mass of all TiO_2_ particles.

### 2.3. Determination of TiO_2_ Content by ICP-AES and DMC

Based on National Food Safety Standard—Standard for the Use of Food Additives (GB 2760-2014), we identified the food categories in which TiO_2_ is used as a food additive. Specific categories surveyed included jams, preserved surface-drying fruit, preserved plum, fried nuts and seeds (TiO_2_ is used in Chinese and European nut products primarily to enhance visual appeal by providing a bright white color, ensuring uniformity, and protecting against discoloration from light exposure. In China, common fried nuts and seeds include fried peanuts, charcoal roasted cashews, chia-chia melon seeds, etc.), cocoa products, chocolate and chocolate products, gum-based candy, other candies excluding gum-based candies, mayonnaise, salad dressing, powdered drinks (powdered drinks include soy milk powder, protein powder, coffee powder, etc.), jelly, and puffed food (puffed food includes potato chips, French fries, potpourri, shrimp chips, etc.). Corresponding products were chosen at random from markets in various provinces across the country. 

The local Centers for Disease Control and Prevention were responsible for testing the levels of TiO_2_ in food and beverages. The testing methods followed the National Food Safety Standard—Determination of Titanium Dioxide in Food (GB 5009.246-2016), which includes the Inductively Coupled Plasma-Atomic Emission Spectrometry (ICP-AES) method and the Diantipyryl Methane Colorimetry (DMC) method (the natural Ti content in food is extremely low, often below the detection limits of standard laboratory testing instruments. The detectable Ti levels in our tests are primarily due to the addition of food-additive TiO_2_.). The detection results under both testing methods showed no statistically significant difference. Specific testing methods and details can be found in Supplementary Material S2.

### 2.4. Dietary Exposure of TiO_2_ and TiO_2_ NPs 

To assess dietary exposure to TiO_2_, we utilized the most recent consumption data available, which came from the Chinese Resident Food Consumption Survey in 2018 by the China National Center for Food Safety Risk Assessment. The survey was conducted in 32 urban survey sites in 18 provinces (autonomous regions and municipalities) in China. The target population was the Chinese population aged 3 years and above. A non-consecutive 3-day, 24 h retrospective method was utilized, aiming to collect comprehensive data on the consumption of various food items for each respondent. After excluding participants with missing or anomalous basic information, a total of 13,585 participants were included in the study, with the consumer group comprising 14.1% of the total. Powdered drinks had the highest average consumption, followed by jelly and puffed foods. The distribution of participant characteristics and consumption levels is provided in Tables S2 and S3.

The following equation is used to calculate daily intake of TiO_2_:

(4) $${EDI}_{i}=\frac{\sum_{k=1}^{n}F_{i,k}\times C_{k}}{{BW}_{i}}$$

*EDI_i_* (mg/kg bw/day) is the estimated daily intake of TiO_2_ of individual *i*. *F_i,k_* is the amount of the *k*th food consumed by individual *i* which was averaged to grams per day over the 3 recording days (g/day). *C_k_* is the average content of TiO_2_ in the *k*th food (mg/g). *BW_i_* is the body weight of individual *i* (kg), and *n* denotes the number of food categories.

Due to the safety concerns surrounding TiO_2_ NPs, we estimated the dietary exposure to TiO_2_ NPs by multiplying the daily intake of TiO_2_ by the mass fraction of NPs.

At present, there are no established recommended intake levels for food-additive TiO_2_ in both domestic and international regulations. Therefore, the Margin of Safety (MoS) approach was used to assess the exposure risk of TiO_2_ and TiO_2_ NPs [25]. The formula for calculating MoS values is as follows [25]:

(5) $$MoS=\frac{NOAEL}{EDI}$$

*MoS* is the Margin of Safety, with values ≥ 100 indicating that the dietary risk of food-additive TiO_2_ is within the acceptable range, while values < 100 indicate that TiO_2_ poses a dietary risk to human health [26]. *NOAEL* is the no observed adverse effect level for TiO_2_, and it was determined to be 1000 mg/kg BW/day based on the results of a reproductive developmental toxicity study [27]. *EDI* is the dietary exposure to TiO_2_, and, in this study, the mean and P95 of exposure were used for the calculation.

### 2.5. Statistical Analysis

The Kruskal–Wallis rank-sum test was employed to analyze differences in daily intake of TiO_2_ and TiO_2_ NPs between groups using the mean. Linear regression analysis was used to assess the association between exposure levels and age. The statistical analyses were conducted by R 4.1.3. All statistical tests were 2-tailed, and differences were considered statistically significant when *p* < 0.05. 

## 3. Results

### 3.1. Size Distribution of Food-Additive TiO_2_ and Proportion of TiO_2_ NPs

**Figure 1:**
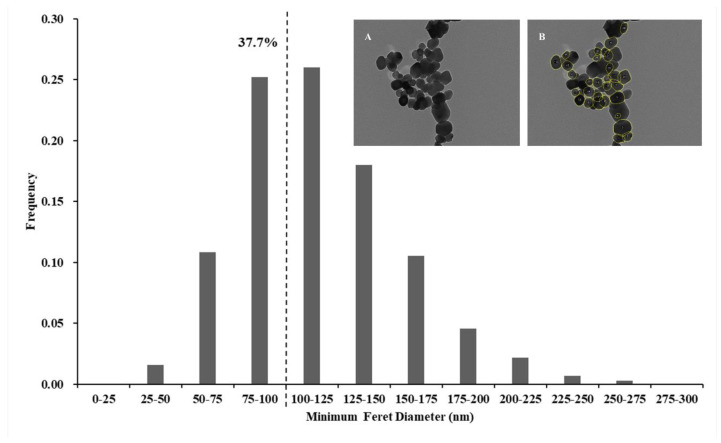
The frequency histogram of particle size distribution of food-additive TiO_2_ samples. The quantitative proportion of TiO_2_ NPs was 37.7%. The TEM images of food-additive TiO_2_ particles showed the primary particle distribution by TEM (**A**) and particle size analysis by ImageJ (**B**) of the TiO_2_ sample.

As depicted in Figure 1, the TEM images revealed an irregular shape for food-additive TiO_2_. The TiO_2_ particles within the size range of 100–125 nm exhibited the highest frequency, comprising 26.0% of the total particle count. Additionally, NPs with a minimum Feret diameter smaller than 100 nm accounted for 37.7%. 

The statistical results in Table 1 showed that a total of 7647 particles were counted from 21 food-additive TiO_2_ samples. The minimum Feret diameters of the particles contained in the 21 samples ranged from 26.8–291.7 nm, with mean values between 103.3–129.1 nm, and an average of 116.4 ± 38.5 nm. The maximum Feret diameters ranged from 33.6–413.6 nm, with mean values between 124.6–159.2 nm, and an average of 143.0 ± 49.8 nm. Based on the mass calculation formula for particles, the estimated mass fraction of nanoparticles was 9.89%.

### 3.2. Content Determination of TiO_2_ in Food Categories

Table 2 presents the content of TiO_2_ in 11 kinds of food categories detected by ICP-AES and DMC. The highest detection rate of TiO_2_ was found in fried nuts and seeds (92.37%), while the lowest was in jam (21.74%). Three categories of food exceeded the permissible limits; namely, cocoa products, chocolate and chocolate products (1.87%), mayonnaise, salad dressing (1.85%), and puffed food (0.34%). The highest content was found in other candies excluding gum-based candies. On average, the content of TiO_2_ measured 186.46 mg/kg, with a median of 0.75 mg/kg. Puffed food emerged as the second highest category, with the average and median values of TiO_2_ content being 146.01 mg/kg and 0.86 mg/kg, respectively. Following closely were cocoa products, chocolate, and chocolate products, with an average content of 140.52 mg/kg for TiO_2_. 

### 3.3. Dietary Exposure of TiO_2_ and TiO_2_ NPs and Differences by Demographic Characteristics

Table 3 shows that the average exposure levels of TiO_2_ and TiO_2_ NPs in the total population were 34.84 μg/kg bw/day and 3.44 μg/kg bw/day, respectively. The P95 values were 161.24 μg/kg bw/day for TiO_2_ and 15.94 μg/kg bw/day for TiO_2_ NPs.

Statistically significant differences were observed among distinct age groups (*p* < 0.001), with the average exposure levels ranking from highest to lowest for children, adolescents, adults, and the elderly. Notably, children displayed the highest intake, with average values of 90.27 μg/kg bw/day for TiO_2_ and 8.93 μg/kg bw/day for TiO_2_ NPs. Gender-based exposure comparisons revealed no significant variations (*p* = 0.846). The daily average intake of males was 33.90 μg/kg bw/day for TiO_2_ and 3.35 μg/kg bw/day for TiO_2_ NPs, while for females, the corresponding figures were 35.68 μg/kg bw/day for TiO_2_ and 3.53 μg/kg bw/day for TiO_2_ NPs. Furthermore, there were significant variations in intake among different provinces (*p* < 0.001), with Inner Mongolia exhibiting the highest exposure levels. The average exposure to TiO_2_ for the Inner Mongolia population was 61.86 μg/kg bw/day, and for TiO_2_ NPs, it was 6.12 μg/kg bw/day. 

Table 4 presents the MoS values for different age groups. Under the NOAEL of 1000 mg/kg bw/day, the calculated lowest MoS was higher than 100, indicating a low safety risk.

### 3.4. Age and Regional Distribution and Food Contribution of TiO_2_ NP Exposure

**Figure 2:**
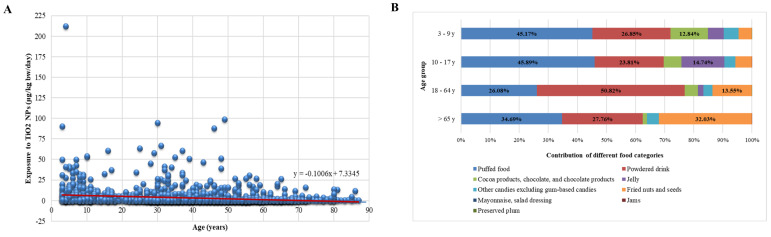
(**A**) The scatter plot of age and TiO_2_ NP exposure in the Chinese population. The linear regression curve equation was given as y = −0.1006x + 7.3345. (**B**) The contribution of different food categories to TiO_2_ NP exposure in various age groups. Puffed food and powdered drinks were identified as the primary dietary sources.

The relationship between age and TiO_2_ NP exposure was examined using linear regression analysis. The results indicated that the regression model was statistically significant (*p* < 0.001). The regression coefficient, which was less than 0, implied a negative correlation between age and TiO_2_ NP exposure. This suggested that, as age increased, the exposure to TiO_2_ NPs tended to decrease (Figure 2A). Based on Figure 2B, it was evident that puffed food and powdered drinks were the primary dietary sources of TiO_2_ NP exposure across various age groups, with contributions ranging from 26.08% to 45.89% for puffed food and 23.81% to 50.82% for powdered drinks. Specifically, for children aged 3-9 years, their main dietary sources included puffed food (45.89%), powdered drinks (23.81%), and jams (14.74%). On the other hand, for adolescents aged 10-17 years, their primary dietary sources were powdered drinks (50.82%), puffed food (26.08%), and fried nuts and seeds (13.55%).

**Figure 3:**
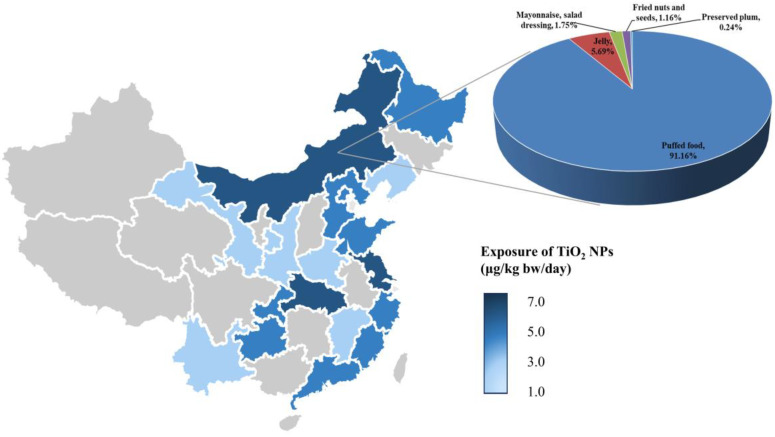
The heat map of TiO_2_ NP exposure in China. Deeper shades of blue on the map indicated higher dietary exposure to TiO_2_ NPs. The pie chart represented the contribution of different food categories to TiO_2_ NP exposure in the Inner Mongolia region.

Figure 3 displays a heat map of TiO_2_ NP exposure in China. Participants residing in Inner Mongolia, Jiangsu, and Hebei regions had higher intakes of TiO_2_ NPs compared to other areas. Among these regions, puffed food contributed the most to TiO_2_ NP exposure for residents of Inner Mongolia, accounting for 91.16% of the exposure. This was followed by jams (5.68%) and mayonnaise, salad dressing (1.75%).

## 4. Discussion

The research findings indicated that in food-additive TiO_2_, the quantitative proportion and the mass fraction of NPs were 37.7% and 9.89%, respectively. Among products available in the Chinese market, other candies, excluding gum-based candies, had the highest content of TiO_2_. The average dietary exposure of the Chinese population to TiO_2_ and TiO_2_ NPs was 34.84 μg/kg bw/day and 3.44 μg/kg bw/day, respectively. Significant differences in dietary exposure were observed among different age groups and regions (*p* < 0.001). Specifically, children aged 3–9 years and residents of Inner Mongolia had the highest average dietary intake. Linear regression analysis revealed a significant negative correlation between TiO_2_ NP exposure and age, with children being at higher dietary exposure risk. The main dietary sources of TiO_2_ NPs were puffed food and powdered drinks. MoS values indicated the dietary exposure risk of TiO_2_ NPs for the Chinese population is acceptable.

The content of TiO_2_ in food or beverages varies among different countries. Domestic research has found the highest concentration of TiO_2_ in dried fig preserves (3409.3 mg/kg), followed by chewing gum and chocolate beans. The content data were significantly higher than the detection data in this study, possibly due to a bias of the limited number of food items and sample size in the study by He et al. [30]. A survey in Turkey revealed high concentrations of TiO_2_ in candy products such as almond nougat (2400 mg/kg), cake decorations (2373 mg/kg), and syrups (2008 mg/kg) [26]. In South Korea, ICP testing of titanium-containing candies found higher levels in chocolate, candies, and chewing gum, with the highest levels being 814 mg/kg, 355 mg/kg, and 42 mg/kg, respectively [31]. TiO_2_ is often used as a whitening agent added to candy coatings, so the TiO_2_ content in candy products typically remains at relatively high levels [32].

Food-additive TiO_2_ produced domestically and internationally was observed by TEM for the particle size distribution and the percentage of NPs. The findings of He et al. [30], which detected NPs in food-additive TiO_2_ in China with quantitative proportion and mass fractions of 34.7% and 10.87%, respectively, were close to the results of our study. The EFSA report in 2016 on the particle size analysis of commercially available TiO_2_ samples under TEM found that the particle diameter ranged from 80–180 nm, with a median diameter of 113 nm. The mass percentage range of nanoparticles was from 0.0% to 3.2%. Approximately 36% of the particles had a diameter smaller than 100 nm [33]. In the 2022 Australia and New Zealand report, it was mentioned that for E171-E, which had the highest nanoparticle content, the median particle size was 104 nm. Approximately 36–45% of the particles had a diameter less than 100 nm [34]. It can be observed that the quantitative proportion of TiO_2_ NPs ranges from 34% to 45%, while the mass fraction may be influenced by the particle crystalline structure and its corresponding density.

TiO_2_ NPs have the potential to accumulate in the body, but their oral bioavailability is low. Following oral ingestion, TiO_2_ NPs are primarily distributed in the liver, spleen, and lungs [35,36]. Studies conducted by Kreyling et al. [36] involved administering TiO_2_ NP suspensions to rats via gavage and observed a slow process of TiO_2_ NP absorption and elimination. Individuals exposed to TiO_2_ NPs over the long term may experience particle accumulation in specific cells and organs. In the study of TiO_2_ NPs given by gavage to rats the highest Ti concentration was detected in the liver, followed by the spleen and small intestine. The oral bioavailability was found to be approximately 0.00021% [37]. Furthermore, research by Chaudhry et al. indicated that the accumulation of TiO_2_ NPs in organs following oral exposure depends on the age of the exposed individuals. Younger individuals have higher intestinal permeability, leading to increased absorption of TiO_2_ NPs [38].

The daily intake of TiO_2_ NPs is influenced by food categories and eating habits in different countries, with higher exposure risks for children. A study investigated the intake of TiO_2_ and its NPs in the Dutch population aged 2 to over 70 years from food, food supplements, and toothpaste. The estimated average intake of TiO_2_ NPs was 0.19 μg/kg bw/day for the elderly, 0.55 μg/kg bw/day for those aged 7–69, and 2.16 μg/kg bw/day for toddlers. Products contributing most to TiO_2_ dietary intake included toothpaste (limited to toddlers) and candies [39]. An Italian study found that the daily intake of TiO_2_ NPs through chewing gum among the European population ranged from 0.28 to 112.40 mg/kg bw/day, with children ingesting more nano-TiO_2_ than adolescents and adults [40]. In 2016, EFSA estimated the dietary intake of NPs at a mass fraction of 3.2%. In a non-brand-loyal exposure scenario, the average daily exposure for infants, adolescents, adults, and the elderly was 0.01 mg/kg bw/day, while children had a daily exposure of 0.18 mg/kg bw/day. Among these groups, 3–9-year-old children had the highest average exposure, with their main dietary sources being candies, sauces, fine bakery wares, and salads [33]. The reasons for the differences in exposure levels between children and adults may be attributed to two factors. First, products containing high levels of food-additive TiO_2_, such as candies, cakes, and other sweets, are highly favored by children. Second, children consume more food than adults when measured in kilograms of body weight [41].

However, in this study, the primary dietary sources of TiO_2_ NP exposure for the Chinese population were found to be puffed food and powdered drinks rather than candies. This difference can be attributed to the food consumption data used in this study. We compared the results of dietary exposure to TiO_2_ for Chinese residents based on the consumption data of the China Health and Nutrition Survey in 2011. Their results indicated significantly higher exposure levels compared to the results of this study. Moreover, sweet foods (such as sugar, chocolate, and cakes) were identified as the largest contributors to TiO_2_ NP intake, accounting for at least 65% of the total intake [30]. A study examining snack consumption patterns in the central and western regions of China observed that approximately 40% of primary and middle school students consume puffed food, with nuts, candies, and chocolates following closely [42]. We reasonably speculate that the consumption of sweet foods in China may have decreased in recent years, while the consumption of puffed food and nut-based products has gradually increased. 

Although this study tested a wide range of food categories where the addition of TiO_2_ is permitted in China and conducted the latest dietary exposure assessment, there are still some limitations to consider. First, the study did not take into account TiO_2_ in food supplements or food packaging materials. However, the impact of such sources is likely to be minimal due to low concentrations and low consumption populations. Secondly, other exposure routes such as dermal exposure and inhalation were not included in the analysis, despite the widespread use of TiO_2_ in cosmetics, personal care products, and other applications. Once the toxic effects and safety limits of TiO_2_ NPs are determined, a multi-route risk assessment will be necessary. Lastly, for the assessment of TiO_2_ intake in the Chinese population, it is important to incorporate updated consumption survey data. This will enable real-time monitoring of exposure dynamics and facilitate timely implementation of population intervention measures and regulation of TiO_2_ as a food additive.

## 5. Conclusions

The study represents the first comprehensive assessment of various food categories permitted to be added with TiO_2_ in the Chinese domestic market, providing the latest exposure assessment for TiO_2_ and its NPs in the Chinese population. The highest content of TiO_2_ was found in other candies excluding gum-based candies. Based on the mass fraction of NPs in food-additive TiO_2_ (9.89%), the estimated average dietary exposure for TiO_2_ and TiO_2_ NPs in the Chinese population was 34.84 μg/kg bw/day and 3.44 μg/kg bw/day, respectively. Children aged 3–9 years and residents of Inner Mongolia face the highest exposure risks. However, the estimated intake of TiO_2_ NPs in this study was far below the levels that could potentially cause harm. 

## Figures and Tables

**Table 1:** Statistical results of particle size of food-additive TiO_2_ samples ^1^.

Sample	Number of Particles	Minimum Feret Diameter (nm)				Maximum Feret Diameter (nm)			
		Mean	SD	Min	Max	Mean	SD	Min	Max
Sample 1	361	103.6	33.8	38.5	238.4	127.9	43.4	50.3	307.5
Sample 2	469	110.5	36.7	27.7	251.5	135.5	48.0	33.6	413.6
Sample 3	224	103.3	39.5	39.8	290.4	128.5	49.9	41.9	328.2
Sample 4	359	123.9	35.2	35.0	279.4	152.4	44.9	45.0	335.6
Sample 5	315	106.8	36.5	29.0	268.7	124.6	46.3	34.0	316.0
Sample 6	331	118.8	38.1	36.3	274.9	146.2	48.7	43.9	350.8
Sample 7	436	112.0	37.5	35.5	265.0	134.9	51.3	40.3	319.6
Sample 8	254	112.8	38.1	51.5	231.7	139.7	49.8	55.1	325.1
Sample 9	300	107.5	34.3	26.8	250.6	133.0	45.4	42.0	379.1
Sample 10	243	111.7	35.7	27.2	227.4	137.7	49.5	43.4	287.5
Sample 11	281	129.1	38.7	32.5	271.3	159.2	49.9	36.5	339.0
Sample 12	392	115.9	39.2	43.6	266.1	138.1	51.0	50.1	342.5
Sample 13	323	111.4	42.0	43.0	222.2	143.7	52.2	52.4	301.7
Sample 14	389	120.0	38.1	40.3	243.5	150.2	48.3	49.5	307.1
Sample 15	384	120.8	36.5	41.6	231.5	149.7	47.3	42.7	321.5
Sample 16	581	119.1	44.2	35.5	266.7	146.2	56.8	46.8	333.4
Sample 17	441	115.0	38.1	44.5	258.8	142.0	46.0	61.8	322.0
Sample 18	286	121.4	37.9	48.5	249.6	153.4	52.8	55.0	330.7
Sample 19	531	121.4	39.3	40.7	255.2	146.4	48.1	56.6	351.3
Sample 20	358	122.9	36.8	35.9	291.7	152.3	47.3	41.8	380.6
Sample 21	389	126.5	38.1	48.5	260.6	153.8	50.4	53.1	365.1
Total	7647	116.4	38.5	26.8	291.7	143.0	49.8	33.6	413.6

^1^ SD: Standard Deviation; Min: Minimum; Max: Maximum.

**Table 2:** Content of TiO_2_ in different food categories ^1^.

Food Category	Maximum Use Level (mg/kg) ^2^	Sample Size	Detection Rate (%)	Exceedance Rate ^4^ (%)	Content of TiO_2_ (mg/kg) ^5^					
					Mean	SD	Median	P95	Min	Max
Jams	5000	46	21.74	0.00	1.07	0.59	1.50	1.50	0.30	2.20
Preserved surface-drying fruit	10,000	27	33.33	0.00	24.68	117.00	1.50	11.68	0.43	610.00
Preserved plum	10,000	18	33.33	0.00	9.12	30.19	1.50	24.91	1.12	130.00
Fried nuts and seeds	10,000	747	92.37	0.00	18.50	308.60	0.30	2.91	0.00	7800.00
Cocoa products, chocolate, and chocolate products	2000	107	86.92	1.87	140.52	529.71	3.32	622.10	0.15	4680.00
Gum-based candy	5000	3	66.67	0.00	2.01	2.24	0.75	4.22	0.69	4.60
Other candies excluding gum-based candies	10,000	84	42.86	0.00	186.46	634.29	0.75	1725.00	0.15	3300.00
Mayonnaise, salad dressing	500	54	37.04	1.85	109.55	377.71	0.30	453.50	0.30	2640.00
Powdered drink	GMP ^3^	122	72.95	0.00	54.82	141.65	9.80	481.25	0.15	783.00
Jelly	10,000	41	34.15	0.00	114.57	294.80	1.10	752.00	0.30	1400.00
Puffed food	10,000	297	69.02	0.34	146.01	862.18	0.86	220.00	0.15	10,200.00
Total	-	1546	75.94	0.26	68.61	491.98	0.42	180.00	0.00	10,200.00

^1^ Considering the left-censored analysis data (i.e., results below the limit of detection (LOD)), an alternative approach was used based on recommendations from the World Health Organization [28] and the European Food Safety Authority [29]. When the non-detection rate of TiO_2_ in food samples was 60% or lower, non-detected values were replaced with 1/2 of the LOD. If the non-detection rate exceeded 60%, the LOD was used to replace the non-detected values. ^2^ The maximum use level for food-additive TiO_2_ can be referenced in the National Food Safety Standard for Food Additive Use (GB2760-2014). ^3^ GMP means use in appropriate amount according to production needs. Through investigation of large enterprises in the powdered drink sector, it was found that only two enterprises in China currently use food-additive TiO_2_ in their powdered drink products, and the maximum use level is 5 g/kg. Therefore, the maximum use level for powdered drinks in this assessment is calculated as 5000 mg/kg. ^4^ The exceedance rate refers to the proportion of the number of food items in which the TiO_2_ content in the surveyed samples exceeded the maximum use level specified in GB2760-2014. ^5^ SD: Standard Deviation; P95: the 95th percentile; Min: minimum; Max: maximum.

**Table 3:** Dietary exposure of TiO_2_ and TiO_2_ NPs by demographic characteristics in the Chinese population (μg/kg bw/day) ^1^.

Subgroups	Dietary Exposure of TiO_2_ (μg/kg bw/day)			Dietary Exposure of TiO_2_ NPs (μg/kg bw/day) ^2^			*p* Value ^3^
	Mean	Median	P95	Mean	Median	P95	
All	34.84	5.47	161.24	3.44	0.54	15.94	
Age							<0.001
3–9 y	90.27	40.51	319.24	8.93	4.01	31.57	
10–17 y	52.24	13.81	136.27	5.17	1.37	13.48	
18–64 y	26.36	4.59	123.91	2.61	0.45	12.25	
>65 y	14.85	4.84	68.83	1.47	0.48	6.81	
Sex							0.846
Male	33.90	5.37	143.98	3.35	0.53	14.24	
Female	35.68	5.61	174.92	3.53	0.55	17.30	
Province							<0.001
Beijing	34.93	5.29	188.39	3.45	0.52	18.63	
Hebei	34.56	5.89	157.49	3.42	0.58	15.57	
Inner Mongolia	61.86	50.57	148.17	6.12	5.00	14.65	
Liaoning	13.80	5.14	65.95	1.36	0.51	6.52	
Heilongjiang	34.20	5.58	132.86	3.38	0.55	13.14	
Jiangsu	58.31	7.63	196.08	5.77	0.75	19.39	
Zhejiang	31.31	3.95	135.41	3.10	0.39	13.39	
Fujian	41.95	6.27	169.05	4.15	0.62	16.72	
Jiangxi	22.27	5.09	100.18	2.20	0.50	9.91	
Shandong	30.68	6.56	137.77	3.03	0.65	13.62	
Henan	13.94	3.51	73.63	1.38	0.35	7.28	
Hubei	58.32	7.32	146.56	5.77	0.72	14.49	
Guangdong	42.85	5.46	239.59	4.24	0.54	23.69	
Chongqing	30.60	6.23	178.46	3.03	0.62	17.65	
Guizhou	30.54	4.20	153.52	3.02	0.42	15.18	
Yunnan	12.44	4.80	44.80	1.23	0.47	4.43	
Shaanxi	28.75	3.93	130.97	2.84	0.39	12.95	
Gansu	19.73	5.26	95.36	1.95	0.52	9.43	

^1^ P95: the 95th percentile. ^2^ The dietary exposure to TiO_2_ NPs was estimated by multiplying the dietary exposure of TiO_2_ by the mass fraction of NPs. ^3^ Differences in exposure by demographic characteristics were analyzed by the Kruskal–Wallis rank-sum test. Significance was set at *p* < 0.05. The results indicated that there were significant differences in population exposure among different ages groups and province groups.

**Table 4:** MoS values calculated based on the exposure estimated in six population groups ^1^.

Age Groups	MoS Values Based on Exposure to TiO_2_		MoS Values Based on Exposure to TiO_2_ NPs	
	Mean	P95	Mean	P95
3–9 y	11,078	3132	111,982	31,676
10–17 y	19,142	7338	193,424	74,184
18–64 y	37,936	8070	383,142	81,633
>65 y	67,340	14,529	680,272	146,843
All	28,703	6202	290,698	62,735

^1^ The Margin of Safety (MoS) values for the mean and P95 of exposure higher than 100 indicated no dietary exposure risk.

## Data Availability

Data is contained within the article.

## Supplementary Materials

The following supporting information can be downloaded at: https://www.mdpi.com/article/10.3390/nano14171427/s1, Table S1. Sources and weighing of food additive TiO_2_ samples. Figure S1. Maximum and minimum Feret diameters measured on TiO_2_ samples under TEM. The particle size of each particle was determined by ImageJ 1.53t software. Table S2. Number and percentage of Chinese consumers by demographic characteristics in 2018. Table S3. Consumption of different food categories in Chinese consumers in 2018 (g/day) ^1^.
