# Supplementary material for: Characterization of Food-Additive Titanium Dioxide and Dietary Exposure to Titanium Dioxide Nanoparticles among the Chinese Population
Source: Nanomaterials (Basel). 2024 Aug 31;14(17):1427. doi: 10.3390/nano14171427 (PMC11397697; doi:10.3390/nano14171427)
Supplement: Supplementary file 1 [file nanomaterials-14-01427-s001.zip › nanomaterials-3054992-supplementary.pdf]

Supplementary Material S1: Sample preparation and particle size detection of food additive TiO_2

1. Preparation of titanium dioxide (TiO_2) dispersion.

Accurately weighed 10.00 ± 0.25 mg of food additive TiO_2 powder and added 6.00 ml of deionized water. An ultrasonic bath (KQ3200B, Kunshan Ultrasonic Instrument Co., Ltd., 150W) was employed for 3 hours, with a 5-minute pause every 30 minutes to prevent water warming. Separately, weighed 1.60 ± 0.02 mg of peptide dispersant powder and dissolved it in 2.00 ml of deionized water. The peptide solution was then added to the ultrasonic TiO_2 suspension to obtain the final TiO_2 dispersion.

Table S1 Sources and weighing of food additive TiO_2 samples

Sample Number	Company	Batch Number	Sample mass/mg	Dispersant dose/mg
Sample1	Jiangsu Hushen Titanium White Technology Co., Ltd	23020701	10.21	1.62
Sample2	Jiangsu Hushen Titanium White Technology Co., Ltd	23010401	10.25	1.62
Sample3	Jiangsu Hushen Titanium White Technology Co., Ltd	22121501	10.25	1.62
Sample4	Jiangsu Hushen Titanium White Technology Co., Ltd	23010801	10.22	1.62
Sample5	Jiangsu Hushen Titanium White Technology Co., Ltd	22122301	10.19	1.62
Sample6	Jiangsu Hushen Titanium White Technology Co., Ltd	22120901	10.05	1.62
Sample7	Jiangsu Hushen Titanium White Technology Co., Ltd	23011101	10.16	1.62
Sample8	Jiangsu Hushen Titanium White Technology Co., Ltd	22120501	10.15	1.62
Sample9	Jiangsu Hushen Titanium White Technology Co., Ltd	22122901	10.25	1.62
Sample10	Jiangsu Hushen Titanium White Technology Co., Ltd	23012901	9.95	1.59
Sample11	Tianjin Duofuyuan Industrial Co., Ltd	2023021601	9.93	1.59
Sample12	Tianjin Duofuyuan Industrial Co., Ltd	2023021501	10.22	1.62
Sample13	Tianjin Duofuyuan Industrial Co., Ltd	2023022301	10.01	1.59
Sample14	Tianjin Duofuyuan Industrial Co., Ltd	2023021901	10.16	1.62
Sample15	Tianjin Duofuyuan Industrial Co., Ltd	2023021801	10.22	1.61
Sample16	Tianjin Duofuyuan Industrial Co., Ltd	2023022401	10.15	1.62
Sample17	Tianjin Duofuyuan Industrial Co., Ltd	2023022001	10.15	1.62

Sample18	Tianjin Duofuyuan Industrial Co., Ltd	2023021301	10.17	1.62
Sample19	Tianjin Duofuyuan Industrial Co., Ltd	2023022201	10.05	1.62
Sample20	Tianjin Duofuyuan Industrial Co., Ltd	2023021101	10.00	1.60
Sample21	China National Center for Food Safety Risk Assessment	2021060301	10.07	1.62

2. Sample preparation and data acquisition of TiO_2 under transmission electron microscope (TEM)

The transmission electron microscope was pretreated with copper mesh substrate. The TiO_2 dispersion was added to the copper net after pretreatment and dried. The size and morphology of the particles were characterized using Transmission Electron Microscopy (TEM, model HT7700, Hitachi). TEM images of TiO_2 were obtained at parameters of accelerating voltage for 10.0 kV, working distance for 500 nm, and magnification for 20,000. The images of each sample were more than 15 pieces, and the number of particles that could be counted with clear edges was more than 200.

3. Data processing and particle size analysis

Particle size was analyzed by ImageJ 1.53t software. In this process, each TiO_2 particle present in the acquired data images was individually measured. Count the number of particles and measure the maximum Feret diameter, minimum Feret diameter, average particle size and so on.

Supplementary Material 2: Determination of TiO_2 content based on "National Food Safety Standard - Determination of Titanium Dioxide in Food" (GB 5009.246-2016)

1. Inductively Coupled Plasma-Atomic Emission Spectrometry (ICP-AES) method

Quantitative measurements were conducted by weighing food or beverage samples and adding a solution containing sulfuric acid for acid digestion to obtain the sample solution. The emission intensity of the sample solution was determined using ICP-AES. The limit of detection (LOD) was 0.3 mg/kg and the limit of quantification (LOQ) was 1.0 mg/kg based on a weighing volume of 0.5 g of the sample and a volume of 50 ml of the sample.

2. Diantipyryl methane colorimetry method

Approximately 5 g of the sample was weighed and subjected to acid digestion. An ascorbic acid solution and a 2-aminoethanol-methane solution were added to prepare the sample solution. The absorbance of the solution was measured at a wavelength of 420 nm using a UV spectrophotometer. The LOD was 1.5 mg/kg and the LOQ was 5.0 mg/kg based on a weighing volume of 0.5 g of the sample and a volume of 50 ml of the sample.

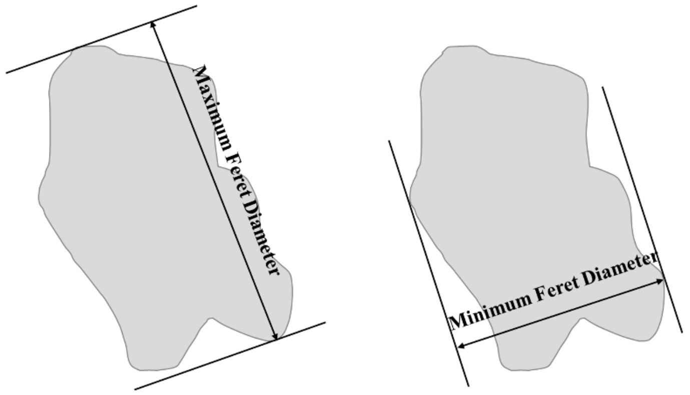

Figure S1 Maximum and minimum Feret diameters measured on TiO_2 samples under TEM. The particle size of each particle was determined by ImageJ 1.53t software.

Table S2 Number and percentage of Chinese consumers by demographic characteristics in 2018

Demographic characteristics	Consumers[1]	Non-consumers[2]	*P* value[3]
**All**	1910(14.1%)	11675(85.9%)	
**Age**			0.308
3 - 9 y	226(16.1%)	1181(83.9%)	
10 - 17 y	143(13.8%)	890(86.2%)	
18 - 64 y	1371(13.7%)	8601(86.3%)	
> 65 y	170(14.5%)	1003(85.5%)	
**Sex**			0.272
Male	901(13.7%)	5693(86.3%)	
Female	1009(14.4%)	5982(85.6%)	
**Province**			0.252
Beijing	39(11.4%)	304(88.6%)	
Hebei	142(21.4%)	522(78.6%)	
Inner Mongolia	12(3.2%)	361(96.8%)	
Liaoning	119(19.7%)	486(80.3%)	
Heilongjiang	134(20.6%)	516(79.4%)	
Jiangsu	204(15.4%)	1123(84.6%)	
Zhejiang	111(15.6%)	600(84.4%)	
Fujian	225(15.5%)	1228(84.5%)	
Jiangxi	63(10.1%)	562(89.9%)	
Shandong	101(21.0%)	379(79.0%)	
Henan	52(9.6%)	490(90.4%)	
Hubei	70(11.2%)	553(88.8%)	
Guangdong	103(14.6%)	602(85.4%)	
Chongqing	221(7.7%)	2643(92.3%)	
Guizhou	42(13.1%)	279(86.9%)	
Yunnan	29(7.1%)	382(92.9%)	
Shaanxi	174(31.0%)	388(69.0%)	
Gansu	69(21.2%)	257(78.8%)	

[1] In this study, the consumers referred to subgroups that consumed specific types of food containing the food additive TiO_2, as reported in the Chinese Resident Food Consumption Survey in 2018.

[2] Non-consumers were those who did not consume any type of food containing food additive TiO_2.

[3] T-test was used for ordinal variable (age) and Chi-square test was used for nominal variables (sex and province). Significance was set at $P < 0.05$.

Table S3 Consumption of different food categories in Chinese consumers in 2018 (g/day) [1]

**Food categories**	**Mean**	**SD**	**Median**	**P95**	**Min**	**Max**
Jams	7.68	18.92	2.39	51.00	0.41	121.35
Preserved surface-drying fruit	-	-	-	-	-	-
Preserved plum	7.68	9.22	5.33	21.10	2.33	35.00
Fried nuts and seeds	12.89	12.16	9.94	35.50	0.08	100.00
Cocoa products, chocolate, and chocolate products	17.68	18.92	10.00	66.67	1.67	83.33
Gum-based candy	-	-	-	-	-	-
Other candies excluding gum-based candies	10.31	11.85	6.67	28.00	1.27	70.67
Mayonnaise, salad dressing	1.22	0.79	1.67	2.10	0.34	2.24
Powdered drink	172.78	211.62	100.00	583.33	1.85	1166.67
Jelly	24.68	25.59	20.00	60.67	3.33	120.00
Puffed food	22.58	19.57	16.67	55.13	0.67	150.00

[1] SD: Standard Deviation; P95: the 95th percentile; Min: Minimum; Max: Maximum.
